# Taxonomic and Phylogenetic Insights into Novel *Ascomycota* from Forest Woody Litter

**DOI:** 10.3390/biology11060889

**Published:** 2022-06-09

**Authors:** Dhanushka N. Wanasinghe, Peter E. Mortimer

**Affiliations:** 1 Center for Mountain Futures, Kunming Institute of Botany, Chinese Academy of Sciences, Honghe 654400, China; 2 Department of Economic Plants and Biotechnology, Yunnan Key Laboratory for Wild Plant Resources, Kunming Institute of Botany, Chinese Academy of Sciences, Kunming 650201, China

**Keywords:** anamorph, Greater Mekong Subregion, *Pleosporales*, teleomorph, Yunnan

## Abstract

**Simple Summary:**

Studies suggest that fungi belonging to *Ascomycota* are sensitive to environmental changes which are disrupting ecosystems globally, with numerous extinction events, imbuing fungal diversity research with a sense of urgency. Thus, it is crucial we assess the diversity of *Ascomycota* across different habitats and substrates, and include programs to seek out novel taxa within *Ascomycota*. Our study provides some insights into the woody-based saprotrophic microfungi in Yunnan, China by introducing two novel *Ascomycota* species. Furthermore, these species were discovered by a 5-year-old boy, thus highlighting how young enthusiasts can be involved in field studies and make a significant impact.

**Abstract:**

While surveying the mycobiomes of dead woody litter in Yunnan Province, China, numerous isolates with affinity to *Pleosporales* (*Dothideomycetes*, *Ascomycota*) were recovered. The present work characterizes two species associated with dead woody twigs found in terrestrial habitats in the Kunming area of Yunnan. The novel taxa were recognized based on a polyphasic approach, including morphological examination and multiple gene phylogenetic analyses (non-translated loci and protein-coding regions). *Neokalmusia* *jonahhulmei* sp. nov. is introduced in *Didymosphaeriaceae* (*Pleosporales*) as a woody-based saprobic ascomycete that possesses multiloculate ascostromata immersed under a black clypeus-like structure, and three-septate, brown, fusiform, guttulate ascospores. *Thyridaria jonahhulmei* (*Thyridariaceae*) is introduced with teleomorphic and anamorphic (coelomycetous) characteristics. The teleomorph has the following characteristics: globose to subglobose ascomata with an ostiolum, a pruinose layer of yellow to reddish- or orange-brown material appearing around the top of the ostiolar necks, and brown, ellipsoid to fusoid, two-to-three-septate, euseptate, rough-walled ascospores; the anamorph features pycnidial conidiomata, phialidic, ampulliform to doliiform, conidiogenous cells, and brown, guttulate, ellipsoidal, aseptate conidia.

## 1. Introduction

Forest ecosystems produce a large quantity of litter in various forms, such as leaves, branches, twigs, inflorescence, and other debris. Plant litter maintains pathways involved in nutrient cycling that are crucial to forest productivity [1,2,3,4,5,6]. A great proportion of total forest carbon (C) is contained in these woody debris, providing habitats for thousands of organisms, including fungi. Fungi play a critical role in the decomposition of woody litter as they are capable of actively decomposing lignin and other recalcitrant components found in these materials. However, comprehensive studies of the fungal taxonomic systematics of woody litter are scarce.

**Figure 1:**
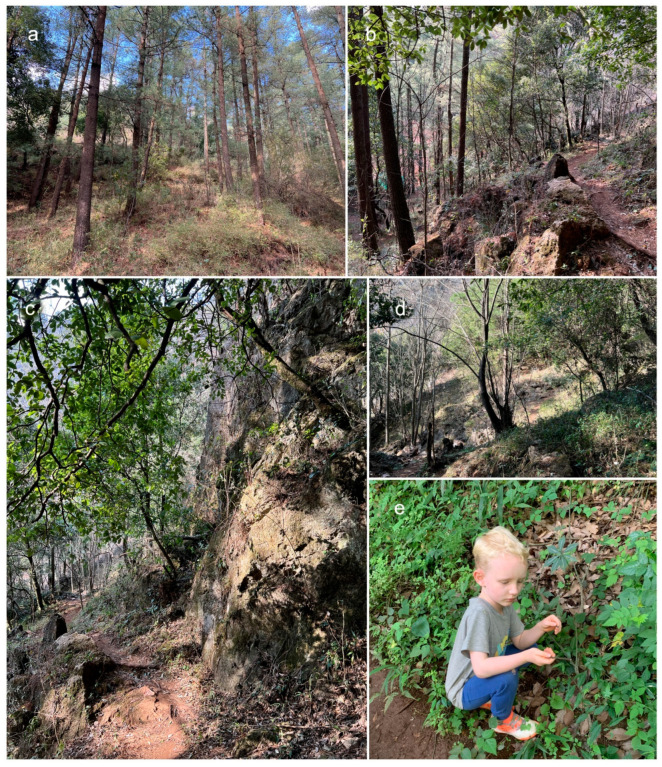
Habitat. (**a**–**d**) Mixed forest (predominantly *Fagaceae* and *Pinus* spp., with *Pinus yunnanensis* being the most abundant tree species); (**e**) collector.

Most of the described woody-based fungal species lack biological and ecological information and reliable taxonomic status. Researchers have paid more attention to economic species, such as those producing edible mushrooms, and less attention to the narrowly distributed microfungal species responsible for the decomposition and recycling of woody material. However, in recent years, there have been numerous studies into woody-based microfungal occurrences in Yunnan Province, leading to reports of higher microfungal diversity, especially in *Dothideomycetes* [7,8,9,10,11,12,13,14,15,16]. At the Centre for Mountain Futures (Kunming Institute of Botany), researchers are investigating microfungal diversity across several substrates in southwest China, including leaf and woody litter, in order to clarify their taxonomy using morphology in conjunction with multigene phylogeny. Accordingly, we have isolated ascomycetes (*Pleosporales*) from woody litter, collected in a *Pinus yunnanensis* forest in Yunnan Province (Kunming), China (Figure 1). This study assesses the systematic categorization of two taxonomic novelties in *Neokalmusia* and *Thyridaria*, assesses morphological characteristics on natural substrates and in cultures, and conducts phylogenetic analyses.

## 2. Materials and Methods

### 2.1. Isolates and Specimens

During fieldwork in the Kunming region, Yunnan, China, characteristic black ascomata/conidiomata on dead woody twigs were collected during the dry season. The local environment features mixed forests (predominantly *Fagaceae* and *Pinus* spp., with *Pinus yunnanensis* being the most abundant tree species) (Figure 1) and a humid temperate climate at elevation 2080 masl. Specimens were stored in Ziploc plastic bags and taken to the laboratory. Isolations were made from single ascospores, according to the methods of Wanasinghe et al. [11]. Voucher specimens were preserved in the herbarium of the Kunming Institute of Botany (KUN-HKAS), and the living cultures were placed in the Culture Collection of Kunming Institute of Botany (KUMCC), Kunming, China. Nomenclatural novelties were deposited in MycoBank as outlined in http://www.MycoBank.org (accessed on 17 March 2022).

### 2.2. Morphological Observations

Examination of external structures of the fungal specimens were made using a Motic SMZ 168 Series stereo-microscope (Motic Asia, Kowloon, Hong Kong). Micro-morphological characters were examined and evaluated following the protocols provided by Wanasinghe et al. [10]. Macroscopic images of colonies were documented using an iPhone XS Max (Apple Inc., Cupertino, CA, USA) with daylight. Photoplates and images were processed using Adobe Photoshop CS6 (Adobe Systems, San Jose, CA, USA).

### 2.3. DNA Extraction, PCR Amplifications and Sequencing

The genomic DNA of each isolate was extracted from scraped mycelia following Wanasinghe et al. [10] using the Biospin Fungus Genomic DNA Extraction Kit-BSC14S1 (BioFlux, Shanghai, China). Primers for PCR amplification used were ITS (internal transcribed spacers) = ITS5/ITS4 [17], LSU (partial 28S large subunit rDNA) = LR0R/LR5 [18,19], SSU (partial 18S small subunit rDNA) = NS1/NS4 [17], *tef*1 (translation elongation factor 1-α) = EF1-983F/EF1-2218R [20,21], and *rpb*2 (RNA polymerase II second largest subunit) = fRPB2-5f/fRPB2-7cR [22]. The PCR protocols were programmed as described in The PCR protocols of SSU, LSU, ITS, and *tef*1 and were programmed as described in Wanasinghe et al. [11]. The PCR amplification condition of *rpb*2 was set as denaturation at 98 °C for 2 min, followed by 35 cycles of denaturation at 98 °C for 10 s, annealing at 52 °C for 10 s, and extension at 72 °C for 20 s, with a final extension step at 72 °C for 5 min. DNA sequencing were performed at a private company for sequencing (BGI, Ltd., Shenzhen, China).

### 2.4. Molecular Phylogenetic Analyses

#### 2.4.1. Sequencing and Sequence Alignment

Sequences generated from SSU, LSU, ITS, *tef*1, and *rpb*2 were first used for BLASTn analyses. Based on BLAST similarities and relevant publications [23,24,25], closely related sequences were downloaded from GenBank (Table 1 and Table 2). Loci were aligned using MAFFT v. 7 [26,27]) under default conditions. Final improvements were made when necessary using BioEdit v.7.0.5.2 software [28].

#### 2.4.2. Phylogenetic Analyses

The single-gene data sets were examined for topological incongruence among loci for members of the analyses. The conflict-free single alignments for each locus were combined into a multi-locus dataset. Concatenated alignment was used to construct maximum-likelihood (ML) and Bayesian (BI) phylogenetic analyses. MrModeltest v. 2.3 [29] was used under the Akaike Information Criterion (AIC) implemented in PAUP v. 4.0b10 to determine the evolutionary models for Bayesian and maximum-likelihood analyses.

The CIPRES webportal [30] was used to execute RAxML [31] and Bayesian analyses [32]. RAxML-HPC2 on XSEDE v. 8.2.10 [30] was used with default parameters and 1000 bootstrap repetitions to construct the ML analysis.

MrBayes analyses were performed setting GTR+I+G as the evolutionary model, with 2 M generations, sampling every 1000 generations, ending the run automatically when standard deviations of split frequencies dropped below 0.01, and with a burnin fraction of 0.25. ML bootstrap values equal or greater than 70% and BYPP greater than 0.95 are given above each node of every tree.

FigTree v1.4.0 program [33] was used to visualize the phylogenetic trees and reorganized in Microsoft Power Point (2007). The finalized datasets and trees were submitted to TreeBASE, submission ID: 29569 (http://purl.org/phylo/treebase/phylows/study/TB2:S29569 (accessed on 17 March 2022)).

## 3. Results

### 3.1. Phylogenetic Analyses

**Figure 2:**
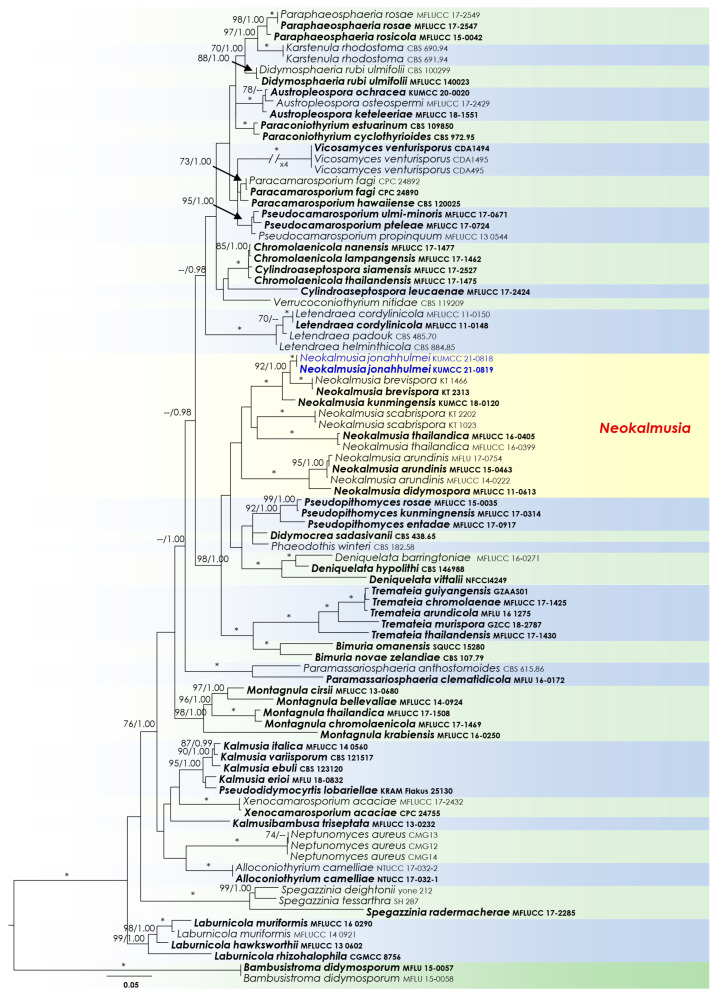
RAxML phylogram generated from combined dataset of partial SSU, LSU, ITS, *tef*1, and *rpb*2 DNA sequence analyses for *Didymosphaeriaceae*. The tree is rooted to *Bambusistroma didymosporum* (MFLU 15-0057, MFLU 15-0058). Bootstrap supports ML (MLB) ≥ 70% and Bayesian posterior probabilities (BYPP) ≥ 0.95 are given as MLB/BYPP above the branches. Branches with an asterisk (*) indicate MLB = 100% and BYPP = 1.00. The newly generated isolates are in blue.

**Figure 3:**
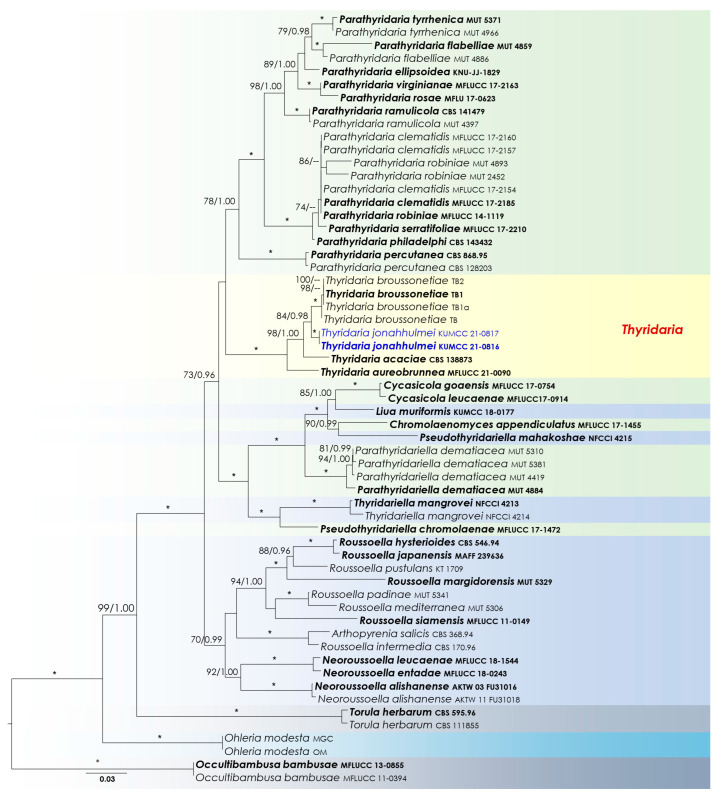
Phylogram generated from RAxML based on a concatenated sequence dataset of partial SSU, LSU, ITS, *tef*1, and *rpb*2 DNA for *Thyridariaceae*. Bootstrap values equal to or greater than 70% for ML (MLB) and Bayesian posterior probabilities (BYPP) ≥ 0.95 are shown at each node (as MLB/BYPP). An asterisk (*) represents branches with MLB = 100% and BYPP = 1.00. The new isolates are show in in blue.

Two analyses were performed in this study. The first is a phylogenetic overview of the genera treated in *Didymosphaeriaceae* (Figure 2), while the remaining alignment represents the genera in *Thyridariaceae* (Figure 3). Other details related to both ML and BI analyses from *Didymosphaeriaceae* and *Thyridariaceae* datasets are presented in Table 3.

### 3.2. Taxonomy

***Pleosporales*** Luttr. ex M.E. Barr, Prodromus to class Loculoascomycetes: 67 (1987)

***Didymosphaeriaceae*** Munk, Dansk botanisk Arkiv 15 (2): 128 (1953)

***Neokalmusia*** Ariyaw. & K.D. Hyde, Fungal Diversity 68: 92 (2014)

**Figure 4:**
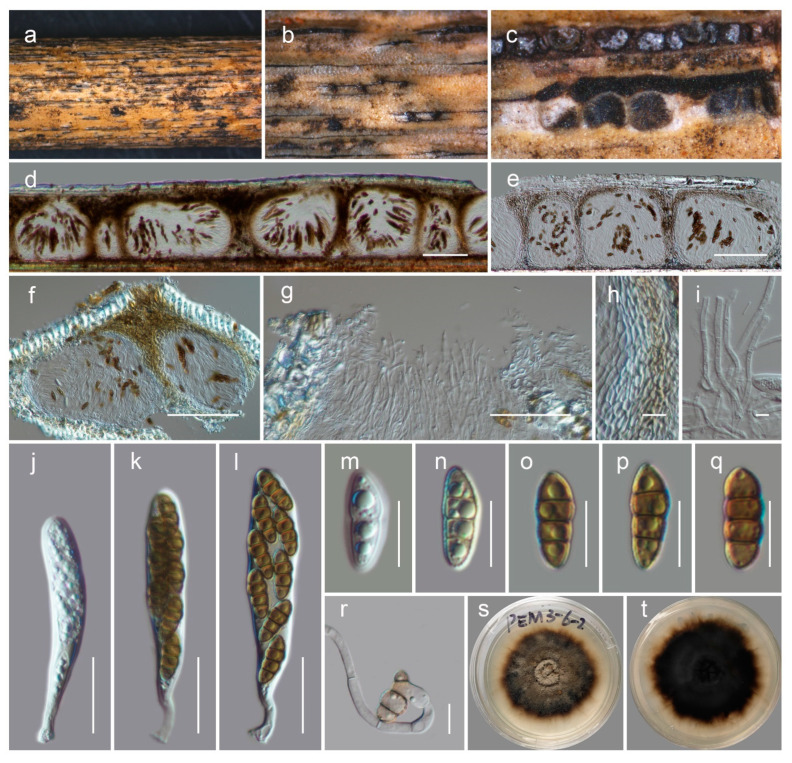
*Neokalmusia jonahhulmei* (HKAS122910, holotype). (**a**,**b**) Ascostromata on the bamboo culms; (**c**) horizontal section of ascostromata; (**d**,**e**) vertical sections of ascomata; (**f**) vertical section through two locules; (**g**) close-up of ostiole; (**h**) peridium; (**i**) pseudoparaphyses; (**j**–**l**) asci; (**m**–**r**) ascospores (r germinated ascospore); (**s**,**t**) colonies on PDA after 21 days. Scale bars, (**d**–**f**) 100 µm; (**g**) 50 µm; (**h**,**m**–**r**) 10 µm; (**i**) 5 µm; (**j**–**l**) 20 µm.

***Neokalmusia jonahhulmei*** Wanas. & Mortimer sp. nov. (Figure 4)

MycoBank: MB843400

*Etymology*: The epithet is derived from Jonah Hulme Mortimer, who is the collector of this fungus.

Holotype: HKAS122910

Saprobic on dead bamboo culms. Teleomorph: *Ascomata* 200–300 μm high × 1200–1500 µm diam. (M = 180 × 1400 µm, *n* = 5), scattered or in groups, immersed under a black clypeus-like structure, composed of host epidermis and fungal mycelium, hemispherical, dark brown to black, multi-loculate. *Locules* 100–170 μm high, 150–280 μm diam. (M = 137.6 × 217.8 µm, *n* = 5), immersed within ascostromata, dark brown to black, subglobose to ampulliform, ostiolate. *Peridium* 10–12 μm wide at the base, 15–30 μm wide at the sides, comprising several layers; outer layers dark brown to brown, with compressed cells of *textura angularis*; inner layers hyaline, with compressed pseudoparenchymatous cells, arranged in *textura angularis*. *Hamathecium* contains 2–3 μm wide, branched, septate, cellular pseudoparaphyses. *Asci* 65–85 × 10–15 μm (M = 72.8 × 11.8 μm, *n* = 15), eight-spored, bitunicate, cylindrical to clavate, curved, short pedicel with slightly furcate ends, apically rounded. *Ascospores* 15–17 × 6–7 μm (M = 15.5 × 6.4 µm, *n* = 30), bi-seriate, overlapping and are initially hyaline, turning brown at maturity, fusiform, three-transversely septate, slightly curved, constricted at the septa, conically rounded at the ends, and smooth-walled, guttulated, without a distinct mucilaginous sheath. Anamorph: undetermined.

Culture characteristics: colonies reaching 4 cm diameter on PDA after 2 weeks at 20 °C. Mycelium dense, circular, slightly raised, smooth on surface and undulated floccose edge. Colony grey at the centre and coffee brown near margin from the top and dark brown at the bottom. Hyphae septate, branched, hyaline, thin, and smooth-walled.

Known distribution: Yunnan, China, on dead woody litter.

Material examined: China, Yunnan, Kunming, Wuhua, 25.131198 N, 102.590770 E, 2080 m, on dead bamboo culms (*Phyllostachys* sp.), 20 March 2021, Jonah Hulme Mortimer, PEM03-6-2-1 (HKAS122910, holotype), ex-holotype culture, KUMCC 21-0818. *ibid*. 25.131178 N, 102.590749 E, PEM03-6-2-4 (HKAS122911), living culture, KUMCC 21-0819.

Notes: The new fungus was collected from dead bamboo culms in Kunming. It is characterized as a typical *Neokalmusia* taxon based on its immersed, hemispherical multi-loculate ascomata under a black clypeus-like structure, bitunicate, clavate, apically rounded asci and bi-seriate, fusiform, brown ascospores with transverse septa [34]. It has a close phylogenetic affinity to *Neokalmusia brevispora* (KT 2313, KT 1466) and *N*. *kunmingensis* (KUMCC 18-0120), with 100 MLB and 1.00 BYPP support values (Figure 2). *Neokalmusia brevispora* and *N*. *kunmingensis* are reported as saprobes from Japan on the *Sasa* sp. and on dead bamboo culms in China, respectively [34,35,36]. These three species are morphologically similar with few dimensional differences in asci and ascospores. *Neokalmusia brevispora* has comparatively larger asci and ascospores (80–118 × 10.5–15 μm; 18–26.5 × 4–7 μm [34]) than *N*. *kunmingensis* (63–77 × 9.6–11.4 μm; 13–15 × 4–5 μm [36]) and our new collection (65–85 × 10–15 μm; 15–17 × 6–7 μm, this study). Further comparison of the ITS regions reveals *Neokalmusia brevispora* and *N*. *kunmingensis* feature nucleotide differences from *N*. *jonahhulmei* of 66/695 (9.5%) and 55/560 (9.8%), respectively. Comparison of the *tef*1 nucleotides of *Neokalmusia brevispora* and *N*. *kunmingensis* with our new strains revealed nucleotide differences of 31/917 (3.4%) and 26/904 (2.9%), respectively. The *rpb*2 region is not available for *Neokalmusia brevispora* and *N*. *kunmingensis* for comparison with our new species. This is the first time *rpb*2 sequence data for a *Neokalmusia* species have been provided (Table 1).

Ariyawansa et al. [34] established *Neokalmusia* to accommodate *N*. *brevispora* and *N*. *scabrispora*, which are characterized by immersed and subglobose to oblong ascomata with multiple perithecia, thin-walled clypeus-like structure, and verrucose ascospores. These two were found on dead culms of *Sasa* and *Phyllostachys* species in Japan, respectively [34,35]. Subsequent studies added four other species *viz*. *N*. *arundinis* (Italy) [37], *N*. *didymospora* (Thailand) [38], *N kunmingensis* (China) [36], and *N*. *thailandica* (Thailand) [37]. In this study, we introduce the seventh species in the genus from *Phyllostachys* species in China. All specimens of these species were obtained from dead culms of Bambusoideae (*Poaceae*) in China, Italy, Japan, and Thailand; therefore, *Neokalmusia* appear to be saprobic on bambusicolous hosts in temperate to tropical environments.

***Thyridariaceae*** Q. Tian & K.D. Hyde, Fungal Diversity 63 (1): 254 (2013)

***Thyridaria*** Sacc., Grevillea 4 (29): 21 (1875)

**Figure 5:**
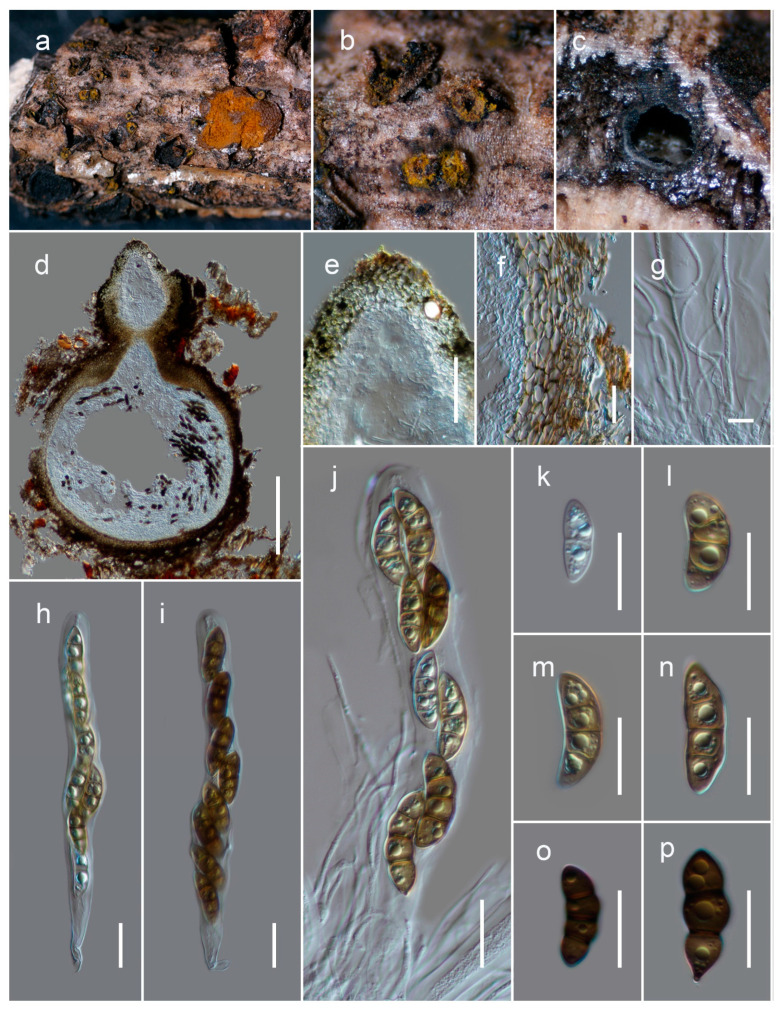
Sexual morph of *Thyridaria jonahhulmei* (HKAS122912, holotype). (**a,b**) Ascomata on the dead woody twigs; (**c,d**) cross section of ascomata; (**e**) close-up of ostiole; (**f**) peridium; (**g**) pseudoparaphyses; (**h**–**j**) asci; (**k**–**p**) ascospores. Scale bars, (**d**) 200 µm; (**e**) 50 µm; (**f,h**–**j**) 20 µm; (**f**,**k**–**p**) 10 µm.

**Figure 6:**
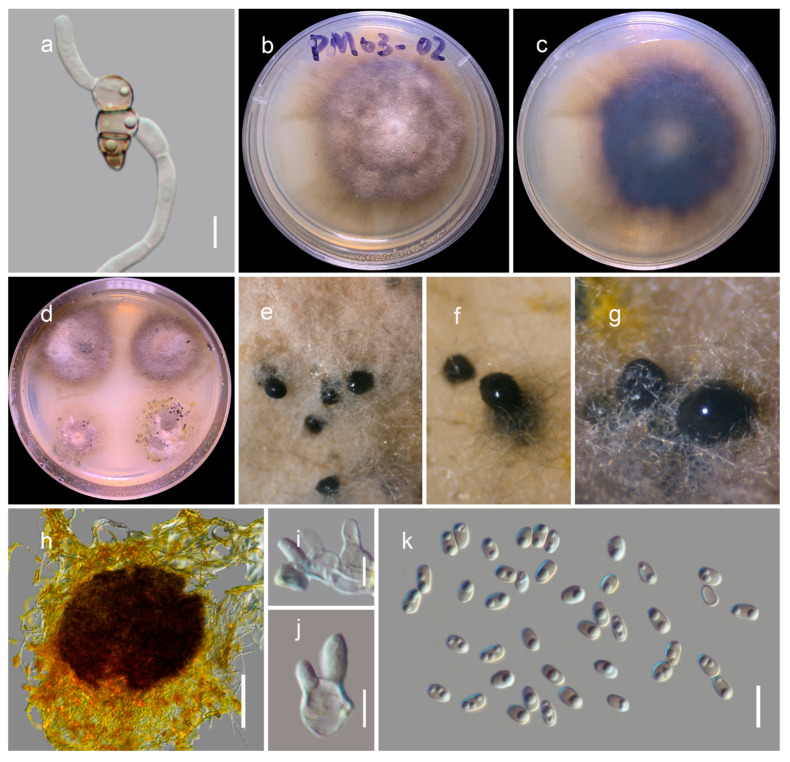
Asexual morph of *Thyridaria jonahhulmei* (KUMCC 21-081, ex-type culture). (**a**) Germinated ascospore (**b**–**d**) colony on PDA (c from the bottom); (**e**–**g**) immersed pycnidia in PDA; (**h**) squashed conidiomata; (**i**,**j**) conidiogenous cells; (**k**) conidia. Scale bars, (**h**) 100 µm; (**i**,**j**) 5 µm; (**k**) 10 µm.

***Thyridaria* *jonahhulmei*** Wanas. & Mortimer sp. nov. (Figure 5 and Figure 6)

MycoBank: MB843401

*Etymology*: The epithet is derived from Jonah Hulme Mortimer, who is the collector of this fungus.

Holotype: HKAS122912

Saprobic on dead twigs of *Fagaceae* sp. Teleomorph: *Ascomata* 550–750 μm high, 200–300 μm diam. (M = 679.3 × 256.4 µm, n = 5), solitary or gregarious, immersed, coriaceous, heavily pigmented, globose to subglobose, ostiolate, yellow to reddish- or orange-brown pruinose layer forming around the apices of the ostiolar necks. Ostiole 100–150 μm long, 40–70 μm diam. (M = 121.1 × 57.8 µm, *n* = 5), central papillate, comprising hyaline periphyses. *Peridium* 15–25 μm wide, wider at the apex (60–70 μm), composed with two layers, with outer stratum comprising pale brown to brown, compressed, thick-walled cells of *textura angularis*, fused with the host tissues, and inner stratum multi-layered and composed with lightly pigmented to hyaline cells of *textura angularis*. *Hamathecium* comprised of 2–2.5 μm wide, branched, septate, cellular pseudoparaphyses, situated between and above the asci, embedded in a gelatinous matrix. *Asci* 150–200 × 18–22 μm (M = 170.2 × 20.1 μm, *n* = 25), eight-spored, bitunicate, fissitunicate, cylindrical to cylindric-clavate, long pedicellate (30–60 μm), and apex rounded with an ocular chamber. *Ascospores* 25–35 × 8–12.5 μm (M = 29.3 × 9.7 µm, *n* = 30), one-to-two-seriate, overlapping, and pale or yellowish brown when young, turning yellowish brown to brown at maturity, narrowly ellipsoid to fusoid, ends narrowly rounded (sometimes pointed), straight or curved, two-to-three-transversely septate, with median euseptum, slightly constricted at the septa, containing several guttules, with surface finely punctate to verruculose. Anamorph: Coelomycetous. *Conidiomata* (1–1.5 mm diam.), pycnidial, scattered or grouped, immersed and heavily pigmented. *Pycnidial* *wall* comprised with several strata, with brown-walled pseudoparenchymatous cells at outer margin becoming hyaline and thin-walled towards the inner conidiogenous cell-layer. *Conidiogenous cells* phoma-like, phialidic, ampulliform to doliiform, hyaline, flexuous, and smooth, with a short collarette. *Conidia* 5.5–8 × 2.5–3.5 μm (M = 6.9 × 3.1 μm, *n* = 50), first hyaline, turning pale brown, one-celled, straight or curved, ellipsoidal, rounded at both ends, thin and smooth-walled, comprising numerous guttules.

Culture characteristics: Colonies spreading on PDA up-to 4 cm diameter after 2 weeks at 20 °C, circular, whitish at the beginning, and becoming slightly raised and greenish-grey after 4 weeks, reverse dark brown. Hyphae septate, branched, hyaline, thin, smooth-walled, producing conidia after six weeks.

Known distribution: Yunnan, China, on dead woody litter.

Material examined: Material examined: China, Yunnan, Kunming, Wuhua, 25.131178 N, 102.590726 E, 2080 m, on dead twigs of woody litter of *Fagaceae* sp., 30 January 2021, Jonah Hulme Mortimer, PM03-2-1 (HKAS122912, holotype), ex-holotype culture, KUMCC 21-0816. *ibid*. 25.131157 N, 102.590708 E, 20 March 2021, PM03-2-3 (HKAS122913), living culture, KUMCC 21-0817.

Notes: Based on multi-gene sequence analyses (SSU, LSU, ITS, *tef*1 and *rpb*2), isolates KUMCC 21-0816 and KUMCC 21-0817 cluster with *Thyridaria acaciae* (CBS 138873), *T*. *aureobrunnea* (MFLUCC 21-0090) and *T*. *broussonetiae* (TB, TB1a, TB1, TB2) with 100 MLB and 1.00 BYPP bootstrap support values (Figure 3). *Thyridaria acaciae* is reported from Tanzania (on *Acacia tortilis*) [39], *T*. *aureobrunnea* from decaying wood in Thailand [40], and *T*. *broussonetiae* from Croatia (*Hippocrepis emerus*), Hungary (*Amorpha fruticosa*), and Italy (*Broussonetia papyrifera*) [41], whereas our new isolate is from dead woody twigs in Yunnan, China. Morphologically *Thyridaria jonahhulmei* has a close affinity to *T*. *aureobrunnea* and *T*. *broussonetiae* in its ascomata, asci, and ascospore characteristics. The asci dimensions of *Thyridaria jonahhulmei* (150–200 × 18–22 μm) are comparatively larger than *T*. *aureobrunnea* (45–61.5 × 7–8.5 µm) and are not significantly different from *T. broussonetiae* (109–183 × 12–19 µm). Comparison of the 546 ITS (+5.8S) nucleotides reveals 43 (7.9%) nucleotide differences between *T*. *aureobrunnea* and *T*. *jonahhulmei*. *Thyridaria aureobrunnea* lacks *tef*1 and *rpb*2 gene regions for comparison. Comparison of the 514 ITS (+5.8S) nucleotides of *Thyridaria broussonetiae* and *T*. *jonahhulmei* reveals 25 (4.86%) nucleotide differences, and in *tef*1 and *rpb*2 there are 8/712 (1.12%) and 18/1025 (1.75%) nucleotide differences, respectively. *Thyridaria acaciae* is known only from its asexual morph, and therefore it is not possible to compare their sexual morphologies. However, the asexual morph of *Thyridaria jonahhulmei* is similar to *Thyridaria acacia*. They both have ampulliform to doliiform conidiogenous cells and pale brown, aseptate conidia. *Thyridaria acacia* has subcylindrical conidia that lack guttules, whereas *Thyridaria jonahhulmei* has ellipsoidal conidia with large guttules. Comparison of the 469 ITS (+5.8S) nucleotides of these two strains reveals 23 (4.9%) nucleotide differences while *tef*1 and *rpb*2 gene regions were unavailable for comparison. Therefore, we recognize these isolates belong to two distinct species [42].

Excluding *Thyridaria acaciae*, *T*. *aureobrunnea* and *T*. *broussonetiae,* our new species resembles *T*. *eminens* (30 × 12 µm [43]), *T*. *koae* (14–21 × 6–9 µm [44]), *T*. *minor* (15–18 × 5–6.5 µm [45]), *T*. *sambucina* (12–15.5 µm [46]), *T*. *subrufa* (12–15 × 4–5 µm [46]), and *T*. *triseptata* (15–18 µm [47]) with its three-septate pigmented ascospores. Among them, *Thyridaria eminens* (collected from dead *Streblus asper* in Philippines [43]) is morphologically closely affiliated with *T. jonahhulmei* by ascospore dimensions. However, all of these species lack molecular data for further phylogenetic characterization.

## 4. Discussion

In this study, we describe and illustrate two new species of microfungi on dead woody litter, *Neokalmusia jonahhulmei* (*Didymosphaeriaceae*) and *Thyridaria jonahhulmei* (*Thyridariaceae*), from Kunming, Yunnan, based on morphological and molecular analyses (Figure 1, Figure 2, Figure 3, Figure 4 and Figure 5). *Neokalmusia jonahhulmei* is introduced with only its sexual characteristics, whereas *Thyridaria jonahhulmei* is accounted for with both asexual and sexual morphological features.

*Didymosphaeriaceae* was introduced by Munk [48], and, given that the family is composed of 33 genera, it is considered one of the most specious families in the order *Pleosporales* [23,25]. Members of *Didymosphaeriaceae* are known to form numerous different types of life modes, including saprobes, pathogens, or endophytes, and can be found both on land or in water [23,49]. We have presented representative sequence data of all currently available genera listed in Hongsanan et al. [23] and Samarakoon et al. [25] for the phylogenetic analyses (except *Barria*, *Curreya*, *Julella,* and *Lineostroma*, for which no DNA-based sequence data were available). Additionally, we included the sequences of *Pseudodidymocyrtis lobariellae* that were introduced by Flakus et al. [50] as a lichenicolous fungus from Bolivia on *Lobariella pallida*. Multi-gene phylogenetic analyses (Figure 2) revealed that *Pseudodidymocyrtis* clusters with *Kalmusia* species, and from the morphological perspective, these two genera appear to have a close resemblance. Therefore, generic delimitation needs defining among these two genera, possibly by using more fresh collections with additional morpho–phylo data.

Even though *Thyridaria* is one of the oldest genera (introduced in 1875) in *Ascomycota*, no exact family was available to accommodate this genus [51]. More than 30 species are included in *Thyridaria* [52]; however, for many of these species, data are lacking illustrations, descriptions, or DNA-based molecular data, leading to ambiguous taxonomic relationships. Therefore, *Thyridaria* species have suffered from uncertain family placement and have been assigned to different families in *Dothideomycetes* at various times [41,53,54,55,56,57,58,59,60,61]. Hyde et al. [51] considered its unique morphology and the phylogenetic placement of *Thyridaria rubronotata* in the *Dothideomycetes* backbone tree and introduced *Thyridariaceae* to accommodate this genus. Recently, Jaklitsch and Voglmayr [41] studied several thyridaria-like genera and constructed a multi-gene analysis to clarify intergeneric taxonomic affinities of *Thyridaria* in the *Pleosporales*. They found that thyridaria-like genera are distributed among at least nine clades of the *Pleosporales*. Barr [61] thoroughly reviewed various descriptions of *Thyridaria* and suggested that a key characteristic of the genus is an ample subiculum surrounding fruiting bodies that are produced under the ascomata wall or in host tissues. Similarly, the intensive study of Jaklitsch and Voglmayr [41] pointed out that this feature is exclusive to delineate *Thyridaria* species from its phylogenetically closely related affiliates. We also observed that our new *Thyridaria* species was characterized with a pruinose layer of yellow to reddish- or orange-brown material forming around the apices of the ostiolar necks. The subiculum of *Thyridaria* species could be an adaptation for its terrestrial-based habitat, and their potential advantages should be further clarified with extensive sampling from both aquatic and non-aquatic habitats.

By contrast, wood-decaying Basidiomycota in tropical China are well studied, which has facilitated a better understanding of global Basidiomycota species diversity and systematics [62,63,64,65,66,67]. Nonetheless, the study of habitat properties and potential lifestyles of woody-based microfungal species is especially important to understanding their functional aspects. The trophic mode of many species can be highly variable, with the ability to switch between mutualistic, pathogenic, and saprotrophic strategies. Pathogenic taxa that cause leaf spots may begin as endophytes, but environmental stressors may necessitate becoming pathogenic or eventually saprotrophic after plant tissue [68,69]. Another example is *Scedosporiosis*, the human disease caused by *Pseudallescheria*/*Scedosporium* complex species (PSC), which can grow saprobically in wood [70,71]. Therefore, owing to their importance in all ecosystems, saprotrophic fungi simply cannot be overlooked in any region.

This study provides some insights into the saprotrophic taxa and contributes knowledge of microfungi associated with woody litter in Yunnan, China by introducing two novel species. Furthermore, these species were discovered by a 5-year-old boy, unassisted by any mycologists at the time of collection, thus highlighting how young enthusiasts can be involved in field studies and make a significant impact.

## Figures and Tables

**Table 1:** Taxa used in the phylogenetic analyses of *Didymosphaeriaceae* and their corresponding GenBank numbers of partial ITS, LSU, SSU, *tef*1, and *rpb*2 sequences. Isolates/sequences in bold were isolated/sequenced in the present study.

Species	Strain	GenBank Accession Numbers				
		ITS	LSU	SSU	*tef*1	*rpb*2
*Alloconiothyrium camelliae*	NTUCC 17-032-1	MT112294	MT071221	MT071270	MT232967	-
*Alloconiothyrium camelliae*	NTUCC 17-032-2	MT112295	MT071222	MT071271	MT232965	-
*Austropleospora keteleeriae*	MFLUCC 18-1551	NR_163349	MK347910	NG_070075	MK360045	MK434909
*Austropleospora ochracea*	KUMCC 20-0020	MT799859	MT808321	MT799860	MT872714	-
*Austropleospora osteospermi*	MFLUCC 17-2429	MK347757	MK347863	MK347974	MK360044	MK434884
*Bambusistroma didymosporum*	MFLU 15-0057	KP761733	KP761737	KP761730	KP761727	KP761720
*Bambusistroma didymosporum*	MFLU 15-0058	KP761734	KP761738	KP761731	KP761728	KP761721
*Bimuria novae-zelandiae*	CBS 107.79	MH861181	AY016338	AY016356	DQ471087	DQ470917
*Bimuria omanensis*	SQUCC 15280	NR_173301	-	NG_071257	MT279046	-
*Chromolaenicola lampangensis*	MFLUCC 17-1462	MN325016	MN325010	MN325004	MN335649	MN335654
*Chromolaenicola nanensis*	MFLUCC 17-1477	MN325014	MN325008	MN325002	MN335647	MN335653
*Chromolaenicola thailandensis*	MFLUCC 17-1475	MN325019	MN325013	MN325007	MN335652	MN335656
*Cylindroaseptospora leucaenae*	MFLUCC 17-2424	NR_163333	MK347856	NG_066310	MK360047	-
*Cylindroaseptospora siamensis*	MFLUCC 17-2527	NR_163337	MK347866	NG_066311	MK360048	-
*Deniquelata barringtoniae*	MFLUCC 16-0271	MH275059	-	MH260291	MH412766	MH412753
*Deniquelata hypolithi*	CBS 146988	MZ064429	-	NG_076735	MZ078250	MZ078201
*Deniquelata vittalii*	NFCCI4249	MF406218	MF622059	MF182395	MF182398	MF168942
*Didymocrea sadasivanii*	CBS 438.65	MH858658	DQ384066	DQ384103	-	-
*Didymosphaeria rubi ulmifolii*	CBS 100299	MH862698	AY642523	JX496124	-	-
*Didymosphaeria rubi ulmifolii*	MFLUCC 140023	KJ436586	NG_063557	KJ436586	-	-
*Kalmusia ebuli*	CBS 123120	KF796674	JN851818	JN644073	-	-
*Kalmusia erioi*	MFLU 18-0832	MN473058	MN473046	MN473052	MN481599	-
*Kalmusia italica*	MFLUCC 14 0560	KP325440	KP325442	KP325441	-	-
*Kalmusia variisporum*	CBS 121517	NR_145165	-	JX496143	-	-
*Kalmusibambusa triseptata*	MFLUCC 13-0232	KY682697	KY682696	KY682695	-	-
*Karstenula rhodostoma*	CBS 690.94	-	GU296154	GU301821	GU349067	GU371788
*Karstenula rhodostoma*	CBS 691.94	LC014559	AB797241	AB807531	AB808506	-
*Laburnicola hawksworthii*	MFLUCC 13 0602	KU743194	KU743196	KU743195	-	-
*Laburnicola muriformis*	MFLUCC 16 0290	KU743197	KU743199	KU743198	KU743213	-
*Laburnicola muriformis*	MFLUCC 14 0921	KU743200	KU743202	KU743201	-	-
*Laburnicola rhizohalophila*	CGMCC 8756	KJ125522	-	KJ125523	KJ125525	KJ125524
*Letendraea cordylinicola*	MFLUCC 11 0150	KM213996	KM214002	KM213999	-	-
*Letendraea cordylinicola*	MFLUCC 11 0148	NR_154118	KM214001	NG_059530	-	-
*Letendraea helminthicola*	CBS 884.85	MK404145	AY016345	AY016362	MK404174	MK404164
*Letendraea padouk*	CBS 485.70	-	GU296162	AY849951	-	-
*Montagnula bellevaliae*	MFLUCC 14 0924	NR_155377	KT443904	KT443902	KX949743	-
*Montagnula chromolaenicola*	MFLUCC 17-1469	NR_168866	NG_070157	NG_070948	MT235773	MT235809
*Montagnula cirsii*	MFLUCC 13 0680	KX274242	KX274255	KX274249	KX284707	-
*Montagnula krabiensis*	MFLUCC 16-0250	MH275070	MH260343	MH260303	MH412776	-
*Montagnula thailandica*	MFLUCC 17-1508	MT214352	NG_070158	NG_070949	MT235774	MT235810
*Neokalmusia arundinis*	MFLU 17-0754	MT649882	MT649880	MT649878	MT663766	-
*Neokalmusia arundinis*	MFLUCC 15-0463	NR_165852	NG_068372	NG_068237	KY244024	-
*Neokalmusia arundinis*	MFLUCC 14-0222	KX965731	KX986344	KX954400	KY271091	-
*Neokalmusia brevispora*	KT 2313	LC014574	AB524460	AB524601	AB539113	-
*Neokalmusia brevispora*	KT 1466	LC014573	AB524459	AB524600	AB539112	-
*Neokalmusia didymospora*	MFLUCC 11-0613	-	KP091435	KP091434	-	-
***Neokalmusia jonahhulmei***	**KUMCC 21-0818**	**ON007043**	**ON007039**	**ON007048**	**ON009133**	**ON009137**
***Neokalmusia jonahhulmei***	**KUMCC 21-0819**	**ON007044**	**ON007040**	**ON007049**	**ON009134**	**ON009138**
*Neokalmusia kunmingensis*	KUMCC 18-0120	MK079886	MK079887	MK079889	MK070172	-
*Neokalmusia scabrispora*	KT 1023	LC014575	AB524452	AB524593	AB539106	-
*Neokalmusia scabrispora*	KT 2202	LC014576	AB524453	AB524594	AB539107	-
*Neokalmusia thailandica*	MFLUCC 16-0405	NR_154255	KY706137	NG_059792	KY706145	KY706148
*Neokalmusia thailandica*	MFLUCC 16-0399	KY706141	KY706136	KY706131	-	-
*Neptunomyces aureus*	CMG12	MK912121	-	-	MK948000	-
*Neptunomyces aureus*	CMG13	MK912122	-	-	MK948001	-
*Neptunomyces aureus*	CMG14	MK912123	-	-	MK948002	-
*Paracamarosporium fagi*	CPC 24890	NR_154318	-	NG_070630	-	-
*Paracamarosporium fagi*	CPC 24892	KR611887	-	KR611905	-	-
*Paracamarosporium hawaiiense*	CBS 120025	JX496027	EU295655	JX496140	-	-
*Paraconiothyrium cyclothyrioides*	CBS 972.95	JX496119	AY642524	JX496232	-	-
*Paraconiothyrium estuarinum*	CBS 109850	JX496016	AY642522	JX496129	-	-
*Paramassariosphaeria anthostomoides*	CBS 615.86	MH862005	GU205246	GU205223	-	-
*Paramassariosphaeria clematidicola*	MFLU 16-0172	KU743206	KU743208	KU743207	-	-
*Paraphaeosphaeria rosae*	MFLUCC 17-2547	MG828935	MG829150	MG829044	MG829222	-
*Paraphaeosphaeria rosae*	MFLUCC 17-2549	MG828937	MG829152	MG829046	MG829223	-
*Paraphaeosphaeria rosicola*	MFLUCC 15-0042	NR_157528	MG829153	MG829047	-	-
*Phaeodothis winteri*	CBS 182.58	-	GU296183	GU301857	-	-
*Pseudocamarosporium propinquum*	MFLUCC 13 0544	KJ747049	KJ819949	KJ813280	-	-
*Pseudocamarosporium pteleae*	MFLUCC 17-0724	NR_157536	MG829166	MG829061	MG829233	-
*Pseudocamarosporium ulmi-minoris*	MFLUCC 17-0671	NR_157537	MG829167	MG829062	-	-
*Pseudodidymocyrtis lobariellae*	KRAM Flakus 25130	NR_169714	NG_070349	NG_068933	-	-
*Pseudopithomyces entadae*	MFLUCC 17-0917		MK347835	NG_066305	MK360083	MK434899
*Pseudopithomyces kunmingnensis*	MFLUCC 17-0314	MF173607	MF173606	MF173605	-	-
*Pseudopithomyces rosae*	MFLUCC 15-0035	MG828953	MG829168	MG829064	-	-
*Spegazzinia deightonii*	yone 212		AB797292	AB807582	AB808558	-
*Spegazzinia radermacherae*	MFLUCC 17-2285	MK347740	MK347848	MK347957	MK360088	MK434893
*Spegazzinia tessarthra*	SH 287	JQ673429	AB797294	AB807584	AB808560	-
*Tremateia arundicola*	MFLU 16 1275	KX274241	KX274254	KX274248	KX284706	-
*Tremateia chromolaenae*	MFLUCC 17-1425	NR_168868	NG_070160	NG_068710	MT235778	MT235816
*Tremateia guiyangensis*	GZAAS01	KX274240	KX274253	KX274247	KX284705	-
*Tremateia murispora*	GZCC 18-2787	NR_165916	MK972750	MK972751	MK986482	-
*Tremateia thailandensis*	MFLUCC 17-1430	NR_168869	NG_070161	NG_068711	MT235781	MT235819
*Verrucoconiothyrium nitidae*	CBS 119209	EU552112	-	EU552112	-	-
*Vicosamyces venturisporus*	CDA1494	MF802825	-	MF802828	-	-
*Vicosamyces venturisporus*	CDA1495	MF802826	-	MF802829	-	-
*Vicosamyces venturisporus*	CDA495	MF802827	-	MF802830	-	-
*Xenocamarosporium acaciae*	CPC 24755	NR_137982	-	NG_058163	-	-
*Xenocamarosporium acaciae*	MFLUCC 17-2432	MK347766	MK347873	MK347983	MK360093	-

**Table 2:** Taxa used in the phylogenetic analyses of *Thyridariaceae* and their corresponding GenBank numbers of partial ITS, LSU, SSU, *tef*1, and *rpb*2 sequences. Isolates/sequences in bold were isolated/sequenced in the present study.

Species	Strain	GenBank Accession Numbers				
		ITS	LSU	SSU	*tef*1	*rpb*2
*Arthopyrenia salicis*	CBS 368.94	KF443410	AY538339	AY538333	KF443404	KF443397
*Chromolaenomyces appendiculatus*	MFLUCC 17-1455	NR_168862	NG_068705	MT214394	MT235770	MT235806
*Cycasicola goaensis*	MFLUCC 17-0754	MG828885	MG829001	MG829112	MG829198	-
*Cycasicola leucaenae*	MFLUCC17-0914	NR_163322	MK347942	MK347833	MK360046	MK434900
*Liua muriformis*	KUMCC 18-0177	MK433599	MK433598	MK433595	MK426798	MK426799
*Neoroussoella alishanense*	AKW 11 FU31018	MK503818	MK503824	MK503830	MK336182	MN037757
*Neoroussoella alishanense*	AKW 03 FU31016	MK503816	MK503822	MK503828	MK336181	MN037756
*Neoroussoella entadae*	MFLUCC 18-0243	MK347786	MK348004	MK347893	MK360065	MK434866
*Neoroussoella leucaenae*	MFLUCC 18-1544	MK347767	MK347984	MK347874	MK360067	MK434876
*Occultibambusa bambusae*	MFLUCC 11-0394	KU940124	KU863113	-	KU940194	KU940171
*Occultibambusa bambusae*	MFLUCC 13-0855	KU940123	KU863112	KU872116	KU940193	KU940170
*Ohleria modesta*	MGC	KX650562	KX650562	-	KX650533	KX650582
*Ohleria modesta*	OM	KX650563	KX650563	KX650513	KX650534	KX650583
*Parathyridaria clematidis*	MFLUCC 17-2154	MT310645	MT214601	MT226712	MT394657	MT394712
*Parathyridaria clematidis*	MFLUCC 17-2157	MT310644	MT214600	MT226711	MT394656	MT394711
*Parathyridaria clematidis*	MFLUCC 17-2160	MT310643	MT214599	MT226710	MT394655	MT394710
*Parathyridaria clematidis*	MFLUCC 17-2185	MT310642	MT214598	NG_070668	MT394654	MT394709
*Parathyridaria ellipsoidea*	KNU-JJ-1829	LC552950	LC552952	-	-	-
*Parathyridaria flabelliae*	MUT 4886	KR014358	KP671720	KT587317	MN605910	MN605930
*Parathyridaria flabelliae*	MUT 4859	KR014355	KP671716	KT587315	MN605909	MN605929
*Parathyridaria percutanea*	CBS 128203	KF322117	KF366448	KF366450	KF407988	KF366453
*Parathyridaria percutanea*	CBS 868.95	KF322118	KF366449	KF366451	KF407987	KF366452
*Parathyridaria philadelphi*	CBS 143432	MH107905	NG_063958	-	MH108023	-
*Parathyridaria ramulicola*	MUT 4397	KC339235	KF636775	MN556311	MN605913	MN605933
*Parathyridaria ramulicola*	CBS 141479	NR_147657	KX650565	KX650514	KX650536	KX650584
*Parathyridaria robiniae*	MUT 2452	MG813183	MG816491	MN556312	MN605903	MN605923
*Parathyridaria robiniae*	MUT 4893	KM355998	MN556328	KM355993	MN605904	MN605924
*Parathyridaria robiniae*	MFLUCC 14-1119	KY511142	KY511141	-	KY549682	-
*Parathyridaria rosae*	MFLU 17-0623	NR_157530	NG_059873	-	-	-
*Parathyridaria serratifoliae*	MFLUCC 17-2210	MT310646	MT214602	NG_070669	MT394658	MT394713
*Parathyridaria tyrrhenica*	MUT 4966	KR014366	KP671740	KT587309	MN605911	MN605931
*Parathyridaria tyrrhenica*	MUT 5371	KU314951	MN556329	KU314952	MN605912	MN605932
*Parathyridaria virginianae*	MFLUCC 17-2163	MT310647	NG_073853	NG_070670	MT394659	MT394714
*Parathyridariella dematiacea*	MUT 4419	KC339245	KF636786	MN556313	MN605905	MN605925
*Parathyridariella dematiacea*	MUT 5310	KU255057	MN556330	MN556314	MN605907	MN605927
*Parathyridariella dematiacea*	MUT 5381	KU314959	MN556331	KU314960	MN605908	MN605928
*Parathyridariella dematiacea*	MUT 4884	MN556317	KP671726	KT587329	MN605906	MN605926
*Pseudothyridariella chromolaenae*	MFLUCC 17-1472	NR_168863	NG_068706	MT214395	MT235771	MT235807
*Pseudothyridariella mahakoshae*	NFCCI 4215	MG020435	MG020438	MG020441	MG023140	MG020446
*Roussoella hysterioides*	CBS 546.94	KF443405	KF443381	AY642528	KF443399	KF443392
*Roussoella intermedia*	CBS 170.96	KF443407	KF443382	KF443390	KF443398	KF443394
*Roussoella japanensis*	MAFF 239636	KJ474829	AB524621	AB524480	AB539114	AB539101
*Roussoella margidorensis*	MUT 5329	KU314944	MN556322	MN556309	MN605897	MN605917
*Roussoella mediterranea*	MUT 5306	KU255054	MN556323	MN556310	MN605898	MN605918
*Roussoella padinae*	MUT 5341	KU158153	MN556325	KU158176	MN605900	MN605920
*Roussoella pustulans*	KT 1709	KJ474830	AB524623	AB524482	AB539116	AB539103
*Roussoella siamensis*	MFLUCC 11-0149	KJ474837	KJ474845	KU872125	KJ474854	KJ474861
*Thyridaria acaciae*	CBS 138873	KP004469	KP004497	-	-	-
*Thyridaria aureobrunnea*	MFLUCC 21-0090	MZ538528	MZ538562	-	-	-
*Thyridaria broussonetiae*	TB	KX650567	KX650567	-	KX650538	KX650585
*Thyridaria broussonetiae*	TB1a	KX650569	KX650569	-	-	-
*Thyridaria broussonetiae*	TB2	KX650570	KX650570	-	KX650540	KX650587
*Thyridaria broussonetiae*	TB1	KX650568	KX650568	KX650515	KX650539	KX650586
***Thyridaria jonahhulmei***	**KUMCC 21-0816**	**ON007041**	**ON007037**	**ON007046**	**ON009131**	**ON009135**
***Thyridaria jonahhulmei***	**KUMCC 21-0817**	**ON007042**	**ON007038**	**ON007047**	**ON009132**	**ON009136**
*Thyridariella mangrovei*	NFCCI 4214	MG020436	MG020439	MG020442	MG020444	MG020447
*Thyridariella mangrovei*	NFCCI 4213	MG020434	MG020437	MG020440	MG020443	MG020445
*Torula herbarum*	CBS 111855	KF443409	KF443386	KF443391	KF443403	KF443396
*Torula herbarum*	CBS 595.96	KF443408	KF443385	KF443387	KF443402	KF443395

**Table 3:** Maximum-likelihood (ML) and Bayesian (BI) analyses results for each sequenced dataset.

Analyses		*Didymosphaeriaceae*	*Thyridariaceae*
Number of taxa		88	59
Gene regions		SSU, LSU, ITS, *tef*1, and *rpb*2	SSU, LSU, ITS, *tef*1, and *rpb*2
Number of character positions (including gaps)		5016	4529
ML optimization likelihood value		−35,672.743881	−30,606.10565
Distinct alignment patterns in the matrix		2249	1796
Number of undetermined characters or gaps (%)		41.88%	19.56%
Estimated base frequencies	A	0.240418	0.249274
	C	0.253351	0.25578
	G	0.270784	0.267476
	T	0.235446	0.227469
Substitution rates	AC	1.561664	1.486771
	AG	3.248718	3.744601
	AT	1.433496	1.706836
	CG	1.323566	1.014483
	CT	7.428045	7.933665
	GT	1.0	1.0
Proportion of invariable sites (I)		0.396829	0.505108
Gamma distribution shape parameter (α)		0.454368	0.442817
Number of generated trees in BI		11,301	2501
Number of trees sampled in BI after 25% were discarded as burn-in		8476	1876
Final split frequency		0.009959	0.009966
The total of unique site patterns		2252	1798

## Data Availability

The datasets generated for this study can be found in the NCBI GenBank, MycoBank, and TreeBASE.
